# Evaluating the Psychometrics of Accelerometer Data for Independent Monitoring of Task Repetitive Practice †

**DOI:** 10.3390/s25216686

**Published:** 2025-11-01

**Authors:** Elena V. Donoso Brown, Rachael Miller Neilan, Fiona Kessler Brody, Jenna Gallipoli, Taylor McElroy, MacKenzie Gough

**Affiliations:** 1 Department of Occupational Therapy, Duquesne University, 600 Forbes Ave, Pittsburgh, PA 15219, USA; 2 Department of Mathematics and Computer Science, Duquesne University, 600 Forbes Ave, Pittsburgh, PA 15219, USA; millerneilanr@duq.edu

**Keywords:** validation, accelerometer, home program, stroke

## Abstract

**Highlights:**

**What are the main findings?**

- In this pilot study, simple measures of duration, velocity, and acceleration from a commercially available wrist-worn accelerometer were shown to be reliable measures of exercise during upper-extremity task practice in a healthy adult population.
- All three measures detected changes in exercise speed, and duration consistently detected differences in exercise time across different tasks.

**What are the implications of the main findings?**

- Study offers preliminary evidence that low-cost wearable sensors can provide reliable, interpretable measures of repetitive task practice in healthy adults.

**Abstract:**

Individuals post-stroke commonly experience impairments in upper extremity function that limit participation in valued activities. Task repetitive practice is an effective intervention strategy, but accurately monitoring adherence and movement quality in home programs remains a challenge. This pilot study investigates the reliability and validity of raw accelerometer data captured by a commercially available, wrist-worn activity monitor to assess upper extremity movement in healthy adults during task repetitive practice. Measures of duration, angular velocity, and acceleration were obtained from activity monitors worn by 25 healthy adults performing four functional tasks under varying conditions. Preliminary results indicate moderate to excellent within-session reliability in these three measures when compared across repeated trials of the same task, with one exception. Across all tasks, the duration measure consistently detected differences in exercise time between sets of 5, 10, and 20 repetitions at a comfortable pace. All three measures differentiated between 10 comfortable repetitions and 10 fast repetitions on three out of four tasks. These findings provide initial psychometric properties in a healthy population and further research is required to determine whether these properties remain robust in the presence of motor impairment. This work represents an early step towards developing approaches for monitoring home exercise programs that support stroke recovery.

## 1. Introduction

Individuals post-stroke often experience impairments in upper extremity function that limit participation in meaningful activities (e.g., [1]). Task repetitive practice or task-oriented training is an evidence-based intervention strategy that involves performing a high volume of repetitions of a particular task or task component to strengthen neural connections and improve motor function [2,3]. Task repetitive practice can be given as part of a home exercise program that individuals post-stroke can complete independently without therapist supervision (e.g., [4]).

Monitoring adherence to home exercise programs has traditionally relied on client self-reports [5], a method which has limitations. Activity monitors provide an alternative means of measuring adherence and quality of exercise [6], both of which are key factors of home programs that are linked to improved recovery outcomes. Body and wrist-worn activity monitors can capture arm function, identify movement types and limitations, and inform traditional standardized assessments [7]. Commonly used measures of arm movement extracted from activity monitors include the ratio of functional to non-functional movement, movement intensity, and activity counts [6]. These measures offer insight into movement properties during daily living and in home practice over time (e.g., [8,9,10,11]).

However, challenges persist in translating accelerometer data to clinical monitoring of task repetitive practice. First, previous studies mostly utilize research grade accelerometers or sensors [12], which may not be accessible in clinical practice or owned by persons post-stroke. This is critical as ease of use and accessibility of equipment impact therapists’ implementation of technology (e.g., [13,14]). Second, while traditional measures of accelerometer data have been used to study arm movement over extended periods of time (e.g., before and after rehabilitation in [15], they are not easily adapted to assess movement during short-term task repetitive practice sessions.

To address this gap, this pilot study aimed to evaluate the psychometric properties of three measures derived from the raw data of a commercially available activity monitor to assess task repetitive practice in healthy, middle-aged adults. A healthy adult sample was used to establish feasibility and generate baseline reliability and validity data, recognizing that translation to post-stroke populations requires further study. The specific research questions addressed in this pilot study were:

(1) Are measures of duration, angular velocity, or acceleration from an activity monitor reliable measures of movement during task repetitive practice?
(2) Are measures of duration, angular velocity, or acceleration from an activity monitor valid measures of movement quality during task repetitive practice?

## 2. Materials & Methods

### 2.1. Participants

Inclusion criteria encompassed healthy adults of age 35–65 without a history of musculoskeletal or neurological conditions that limit normal movement. This age range was selected because it encompassed a group of healthy adults available in the setting where data collection occurred. In addition, this convenience sample was less likely to have co-morbidities (e.g., rotator cuff injury) that would exclude participation. No incentives were given, and recruitment was designed to avoid coercion of participants familiar with the primary investigator. Twenty-five adults (9 males, 16 females) were recruited for this study. The mean age of the participants was 47.17 years (SD = 10.17), with 22 of the 25 individuals identified as right-hand dominant.

### 2.2. Outcomes Measures

The outcome measures were duration, total angular velocity, and standard deviation of acceleration. These measures were calculated from raw data collected by commercially available Microsoft Bands [16] and a custom mobile application run through a Microsoft phone. Raw data were collected at 62 Hz and included angular velocity (degrees per second) and acceleration (meters per second^2^) along the x, y, and z axes. These measures were selected as they could approximate meaningful information for task repetitive practice such as the time it took to complete a set (duration), the typical speed of rotational movement during practice (total angular velocity), and variation in movement forces (standard deviation of acceleration), which have been related to functional use [17,18].

### 2.3. Procedures

The procedures received approval from the institutional IRB and all participants completed written informed consent. Participants were screened to assess bilateral upper extremity range of motion and motor coordination to ensure there were no limitations that could impact the study.

Participants completed five functional tasks (Table 1) in a single session with activity monitors worn on each wrist. Each task was repeated across four different conditions (5, 10, and 20 repetitions at a comfortable pace, 10 repetitions at a fast pace) and one repeated condition (10 repetitions at a comfortable pace). Task order was randomized and subsequently conditions were randomized for each participant. Thirty-second rest periods were given in between each condition in a task.

### 2.4. Data Processing and Analysis

Only data from the dominant-side accelerometer was used as our outcomes did not require bilateral data. All accelerometer data was saved as .csv files, sorted by participant and activity, and imported into MATLAB [19] for analysis. Analyses were performed using the custom MATLAB program [9]. Readers can find the MATLAB program at https://osf.io/wz5my/. The program parsed the raw data into six vectors containing the absolute value of the angular velocity and acceleration in the x, y, and z planes, respectively, and generated six graphs displaying these values over the entire length of time represented by the data set. Start and stop times for each trial were visually identified from these graphs as transitions from rest (zero velocity) to activity (non-zero velocity) and vice versa. This process was supported generally by the required rest period between the trials. Data between the start and stop times was analyzed and excluded rest periods. In the few instances when start and stops could not clearly be identified, the data for that task were not included. This occurred across five participants and a total of 31 trials out of the 500 (6.2%) trials analyzed.

The MATLAB program output measures of duration, angular velocity, and acceleration for each trial. Duration (seconds) was calculated as the difference between start and stop times. The average, standard deviation, max, and min of angular velocity (degrees/s) and acceleration (m/s^2^) for each trial were also calculated. To answer the specific research questions, the following measures were used: duration, average total angular velocity $\left(\bar{V_{x}+V_{y}+V_{z}}\right)$, and the total standard deviation of acceleration ($\sigma_{x}+\sigma_{y}+\sigma_{z})$. The average total angular velocity was chosen as it reflects typical rotational speed needed to complete a task, providing an index of functional performance. Angular velocity has been shown to be an important variable in discriminating movement profiles of healthy individuals and those who have had a stroke, as well as between different stroke impairment levels [17]. Standard deviation of acceleration was chosen because it captures variability in movement forces. Standard deviation of acceleration has been shown to positively correlate with functional performance of the affected upper extremity post-stroke [18].

### 2.5. Statistical Analysis

To test the within-session reliability of our outcome measures, an Intraclass Correlation Coefficient (ICC) was calculated for each task using the repeated trials of 10 repetitions at a comfortable pace completed within the same session. We selected a model that accounted for two-way mixed effects, mean of k measurements, absolute agreement for angular velocity and standard deviation of acceleration (3, k) and two-way mixed effects, with single measurement (3, 1) for duration. These decisions were guided by recommendations from [20] to account for the way the band was a single fixed-rate rater and the two sets of data points being non-random. The type of model was selected based on whether the data were a single measurement or an average of measurement points. Thresholds for reliability were ICC > 0.9 as excellent, between 0.9 and 0.75 as good, and between 0.75 and 0.5 as moderate [21].

To determine if our outcome measures could assess movement parameters important to monitoring upper extremity use, a repeated measures ANOVA or Friedman’s test (depending on normality) was performed for trials of 5, 10, and 20 repetitions of each task at a comfortable pace. For this test α = 0.004 using Bonferroni correction, sphericity was checked, and effect size with partial eta squared or Kendall W was completed. Additionally, paired T-tests were performed to compare the outcome measures from trials of 10 repetitions at a comfortable and 10 repetitions at a fast pace. For the paired T-tests, statistical significance was set at α = 0.004 using a Bonferroni correction for 12 multiple tests.

## 3. Results

With one exception, the accelerometer data showed moderate to excellent within-session reliability (ICC values between 0.52–0.90) when comparing measures of duration, angular velocity, and acceleration across repeated conditions (Table 2). The measure with poor reliability (ICC < 0.5) was duration in the writing task (ICC = 0.31).

Across all five tasks, measures of duration differed significantly between sets of 5, 10, and 20 repetitions at a comfortable pace (*p* < 0.001) (Table 3). Measures of angular velocity and acceleration did not vary significantly between sets of 5, 10, and 20 comfortable repetitions, except for the writing task. The writing task yielded significantly lower measures of angular velocity as the number of repetitions increased from 5 to 20 (*p* < 0.001) which was also reflected in the effect size. The effect size also was large in writing for acceleration although not statistically significant.

All three measures showed statistically significant differences between 10 comfortable repetitions and 10 fast repetitions of the same task (Table 4), except for the writing task which showed no significant difference in total angular velocity and acceleration and the shoe tying task which showed no difference in duration.

## 4. Discussion

This pilot study examined the psychometrics of three simple measures of duration, angular velocity, and acceleration calculated from raw accelerometer data collected via a commercially available activity monitor worn by healthy adults during upper extremity task repetitive practice. Acceptable levels of within-session reliability were found across most measures and tasks. Duration consistently captured changes in exercise time (5, 10, and 20 repetitions); however, significant changes were not seen in angular velocity and acceleration except in the writing task. The absence of differences in these two variables across repetition counts suggests that individuals with typical movement patterns maintained consistent speed and smoothness, as would be expected in the absence of fatigue [8]. All three measures differentiated between two exercise speeds (comfortable vs. fast pace) in all but one task per measure. These findings provide preliminary evidence that simple measures from commercially available accelerometers can capture meaningful variations in task-repetitive practice under controlled conditions in healthy adults.

Compared to previous work [10,23], this study used straightforward measures of movement that can be easily interpreted. For example, the reliability findings suggest that some tasks may be more consistent than others. Specifically, the writing task exhibited variability possibly due to learning how to write or increased comfort in writing the specific name between trials. Additionally, the validity findings suggest that angular velocity may be a useful indicator of upper extremity fatigue, consistent with prior work [24], and may explain the reduced velocity observed in the writing task. This was the only task that showed a change in both speed and duration with an increasing number of repetitions, but specifically a decrease in speed across the increased number of repetitions. Also of note was that the writing task did not demonstrate changes in angular velocity or acceleration when comparing typical versus fast. This could be because “fast” was not specifically defined or because the directions indicated that the name still needed to be legible which placed a limit on the amount of speed the participants could increase and meet this direction.

This study has several limitations that should be considered when interpreting the findings. Most notably, the sample population included 25 healthy, middle-aged adults representing only a portion of the age range of the population post-stroke. This sample provided a controlled setting to establish feasibility and examine preliminary psychometric properties; however, it limits translation to individuals post-stroke who are older and may present with paresis, fatigue, orthopedic issues, and compensatory movements. Moreover, the small sample size (n = 25) limited the precision estimates of ICC values measuring within-session reliability. Additionally, the convenient nature of this sample left an imbalance of males and females limiting comparisons. Future studies should replicate these analyses with larger and more diverse samples and include collection of data over a multi-day period to allow for accurate estimation of test-retest reliability.

Other limitations of this study include the use of an older-generation activity monitor, restriction of our analyses to the dominant hand only, and visual analysis to identify starts and stops. The Microsoft band used in this study has been discontinued and is no longer supported by its manufacturer. Although the device was sufficient for the purpose of our pilot study, newer wearable devices offering higher sampling rates and improved sensor fidelity should be considered in future studies. Further, only data from the dominant wrist were analyzed, which reduced our ability to assess additional variables and consider the role of each extremity, dominant and non-dominant. Additionally, the use of visual inspection to identify the starts and stops could have introduced error into the analysis, and in the future should be replaced with automated identification utilizing thresholds.

Future directions for this work include replication studies with healthy persons and persons post-stroke to determine if in a larger sample these variables remain reliable and valid. Additionally, including both healthy individuals and persons post-stroke would allow for comparison between the groups and exploration of known groups’ validity. Further, validation with gold standard upper extremity measures would be supportive in future work. It would also be beneficial to consider the reliability of these variables across sessions as well as within sessions. Future studies would also assess whether measures of duration, angular velocity, and acceleration remain reliable and valid in the presence of motor impairments and compensatory movements. Some indications of feasibility have been found in previous secondary analyses (e.g., [9]); however, further replication in a controlled environment is needed. In addition, continued standardization of tasks with considerations for including a metronome to determine speed and a device-agnostic pipeline would be important inclusions in future work. Finally, future investigations can also consider other metrics known to be important in identifying movement patterns of persons post-stroke (e.g., peak angular velocity) [17] or bilateral use [25].

## 5. Conclusions

This initial investigation highlights the potential for simple outcome measures captured from raw accelerometer data to be valuable in monitoring repetitive task practice in healthy adults. This pilot study serves as a springboard for further investigation of these measures with persons post-stroke.

## Figures and Tables

**Table 1:** Visuals and instructions for each task.

Task	Task Image	Task Description
Picking up a cup	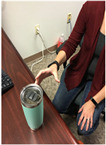	Participants were seated for this task. Hands were in their lap. Then using the dominant hand, the participants picked up the cup, brought it to the mouth and placed the cup back on a table. Participants ended with their hand in their lap.
Handwriting	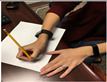	Participants wrote “Taylor Swift” with their dominant hand while seated. This name was chosen as it has the average length of a name, is a recognizable name, and is easy to spell. The same pen was used by all participants. For fast condition name had to be legible.
Putting a letter in a mailbox	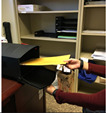	While seated an arm’s length (with elbow extended) from the mailbox, participants opened the mailbox with the non-dominant hand, placed a letter inside with the dominant hand, and then closed the mailbox with non-dominant hand.
Tying a shoe	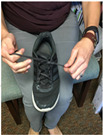	Participants tied the shoe placed on their lap and then untied the shoe using both hands. Participants were instructed not to double knot the shoe and to keep the shoe at midline.

**Table 2:** ICC values by task and outcome measure.

Task	n	Duration (3, 1) ICC	Total Angular Velocity (3, k) ICC	Standard Deviation of Acceleration (3, k) ICC
Letter	24	0.82 95% CI [0.61, 0.92]	0.95 95% CI [0.88, 0.98]	0.95 95% CI [0.89, 0.98]
Shoe	24	0.80 95% CI [0.59, 0.91]	0.92 95% CI [0.80, 0.96]	0.91 95% CI [0.79, 0.96]
Cup	23	0.71 95% CI [0.44, 0.87]	0.94 95% CI [0.85, 0.97]	0.89 95% CI [0.74, 0.95]
Writing	24	0.31 95% CI [−0.10, 0.63]	0.92 95% CI [−0.82, 0.97]	0.86 95% CI [0.69,0.94]

Note: 95% CI indicates 95% confidence intervals for ICC value.

**Table 3:** Repeated measures ANOVA results across outcome measures and tasks.

**Duration (s)**						
		**5 Reps**	**10 Reps**	**20 Reps**	**Test**	**Effect**
**Task**	**n**	**M (SD)**	**M (SD)**	**M (SD)**	**Statistic**	**Size (ηp^2^)**
Letter	24	20.88 (6.32)	39.40 (12.03)	76.72 (14.85)	44.33 ^*	0.92 ^
Shoe	24	49.95 (21.42)	87.88 (18.42)	164.84 (41.34)	40.33 ^*	0.84 ^
Cup	23	17.57 (3.13)	35.67 (6.76)	66.87 (11.69)	576.59 *	0.96
Writing	24	30.73 (4.65)	52.81 (7.07)	100.55 (13.85)	770.84 *	0.97
**Total Angular Velocity (d/s)**						
		**5 reps**	**10 reps**	**20 reps**	**Test**	**Effect**
**Task**	**N**	**M (SD)**	**M (SD)**	**M (SD)**	**Statistic**	**Size (ηp^2^)**
Letter	24	142.62 (43.02)	147.77 (37.96)	146.61 (32.46)	0.40	0.02
Shoe	24	155.56 (32.66)	155.52 (31.37)	160.68 (30.93)	1.85	0.08
Cup	23	110.00 (4.55)	108.46 (4.76)	111.35 (4.50)	0.79	0.04
Writing	24	43.42 (11.83)	37.14 (8.90)	32.75 (9.76)	26.53 *	0.54
**Standard Deviation of Acceleration (m/s^2^)**						
		**5 reps**	**10 reps**	**20 reps**	**Test**	**Effect**
**Task**	**N**	**M (SD)**	**M (SD)**	**M (SD)**	**Statistic**	**Size (ηp^2^)**
Letter	24	1.56 (0.06)	1.56 (0.06)	1.55 (0.05)	0.34	0.01
Shoe	24	1.48 (0.05)	1.47 (0.04)	1.48 (0.04)	0.14	0.006
Cup	23	1.55 (0.03)	1.55 (0.03)	1.55 (0.03)	1.22	0.05
Writing	24	1.46 (0.04)	1.46 (0.04)	1.45 (0.03)	8.11 ^	0.17 ^

Note: M indicates mean value, SD indicates standard deviation, and Test statistic indicates either an ANOVA F-test statistic or a chi square from Friedman’s test. All tests demonstrated adequate sphericity. Partial eta squared (ηp^2^) was used to calculate effect size for the ANOVA and can be cautiously interpreted with the following guidelines a ηp^2^ = 0.01 as a small effect, ηp^2^ = 0.06 as a medium effect, and ηp^2^ = 0.14 as a large effect [22]. Kendell W interpretation should be in accordance with correlation-based effect sizes where small is 0.10, medium is 0.3 and large is 0.5 [21]. ^ Indicates that Friedman’s test with effect size calculated with Kendall’s W was completed as a non-parametric equivalent. * Denotes *p*-value < 0.001.

**Table 4:** Mean change between conditions 10 at a fast pace and 10 at a comfortable pace.

				**Duration (s)**			
		**10 Comfortable**	**10 Fast**	**Mean Difference**			
**Task**	**N**	**M (SD)**	**M (SD)**	**M (SD)**	**T-Statistic**	** 95% CI **	** Cohen’s d **
Letter	24	39.40 (12.04)	29.54 (7.39)	−9.85 (12.08)	−4.00 *	95% CI [−14.96, 4.76]	−0.96
Shoe	24	87.88 (18.42)	78.97 (25.17)	−8.91 (19.52)	−2.24	95% CI [−17.15, −0.67]	−0.39
Cup	23	35.67 (6.76)	23.95 (6.76)	−11.72 (7.29)	−7.71 *	95% CI [−14.87, −8.57]	−1.92
Writing	23	53.26 (6.87)	45.71 (8.87)	−7.54 (9.75)	−3.71 *	95% CI [−11.76, −3.33]	−0.95
**Total Angular Velocity (degrees/s)**							
		**10 Comfortable**	**10 fast**	**Mean Difference**			
**Task**	**N**	**M (SD)**	**M (SD)**	**M (SD)**	**T-Statistic**	** 95% CI **	** Cohen’s d **
Letter	24	147.52 (37.95)	185.73 (37.62)	38.22 (37.77)	4.96 *	95% CI [22.26, 54.17]	1.01
Shoe	24	155.52 (31.37)	179.43 (35.74)	23.91 (28.04)	4.18 *	95% CI [12.07, 35.75]	0.71
Cup	23	108.46 (22.82)	147.54 (27.27)	39.08 (22.58)	8.30 *	95% CI [29.32, 48.85]	1.54
Writing	23	36.93 (9.04)	42.90 (15.53)	5.97 (11.58)	2.47	95% CI [0.96, 10.98]	0.42
**Total Standard Deviation of Acceleration (m/s^2^)**							
		**10 Comfortable**	**10 fast**	**Mean Difference**			
**Task**	**N**	**M (SD)**	**M (SD)**	**M (SD)**	**T-Statistic**	** 95% CI **	** Cohen’s d **
Letter	24	1.56 (0.65)	1.65 (0.12)	0.09 (0.09)	4.82 *	95% CI [0.05, 0.13]	0.83
Shoe	24	1.47 (0.04)	1.51 (0.05)	0.04 (0.03)	5.35 *	95% CI [0.02, 0.05]	0.72
Cup	23	1.55 (0.05)	1.61 (0.03)	0.06 (0.05)	5.79 *	95% CI [0.04, 0.08]	1.37
Writing	23	1.46 (0.04)	1.47 (0.04)	0.01 (0.03)	1.81	95% CI [−0.01, 0.03]	0.33

Note: M indicates mean value, SD indicates standard deviation, mean difference values represent the mean change in each of the three measures when comparing 10 repetitions at a fast pace to 10 repetitions at a comfortable pace. The three measures are duration (s), total angular velocity (d/s), and standard deviation of acceleration (m/s^2^). Paired T-tests were applied to determine if there was a significant change in each of the three measures for these two conditions. * Indicates statistical significance *p* < 0.004. Cohen’s d effect sizes can be interpreted with d = 0.20 as small, d = 0.50 as medium/moderate and, d = 0.80 as large [21].

## Data Availability

The raw data supporting the conclusions of this article will be made available by the authors on request.
